# Supplementary material for: 7-Acetoxycoumarin Inhibits LPS-Induced Inflammatory Cytokine Synthesis by IκBα Degradation and MAPK Activation in Macrophage Cells
Source: Molecules. 2020 Jul 8;25(14):3124. doi: 10.3390/molecules25143124 (PMC7397006; doi:10.3390/molecules25143124)

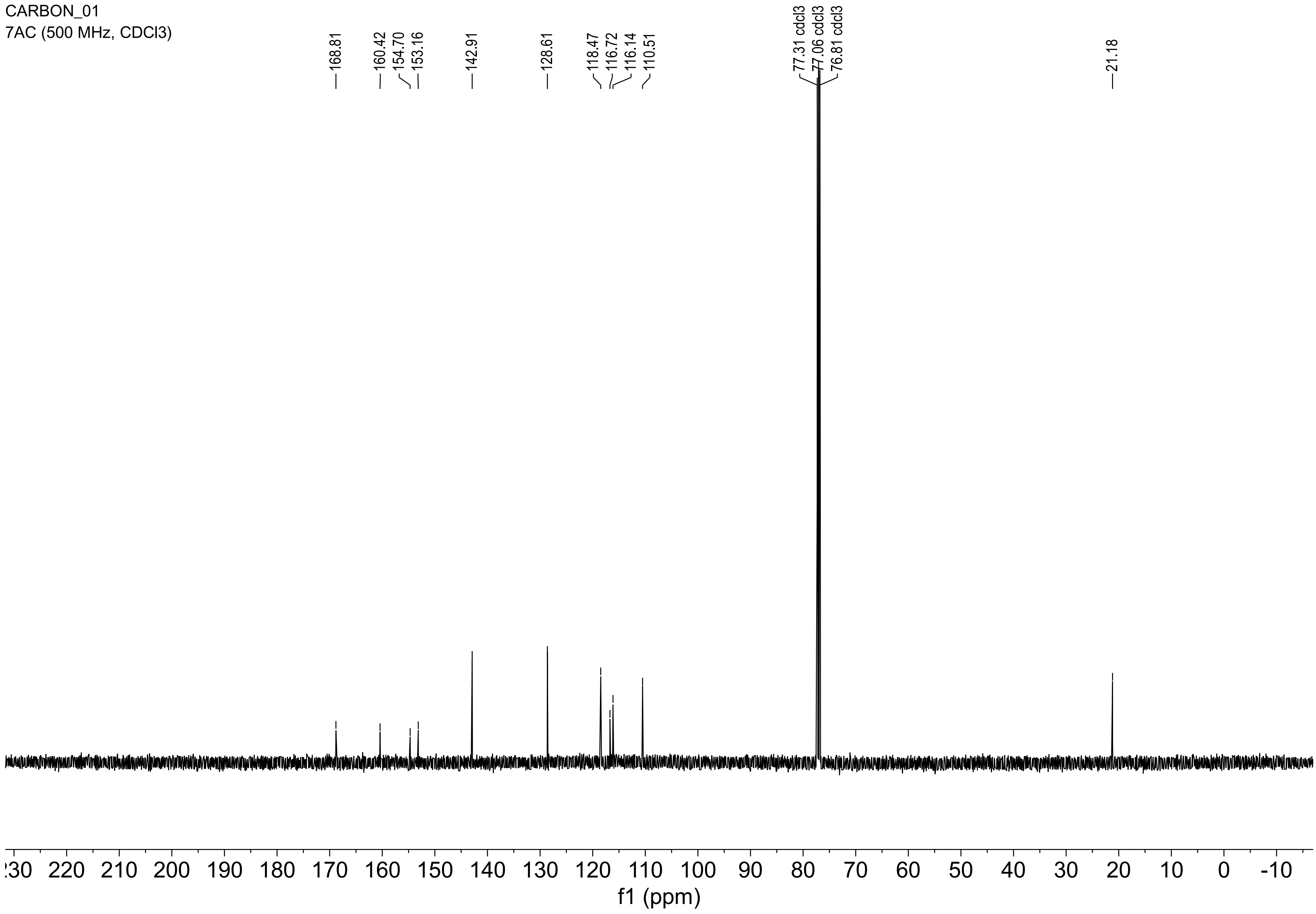

CARBON_01
7AC (500 MHz, CDCl3)
168.81
160.42
154.70
153.16
142.91
128.61
118.47
116.72
116.14
110.51
77.31 cdcl3
77.06 cdcl3
76.81 cdcl3
21.18
230 220 210 200 190 180 170 160 150 140 130 120 110 100 90 80 70 60 50 40 30 20 10 0 -10
f1 (ppm)

^{1}H NMR (500 MHz, Chloroform-d) δ 7.71 (d, J = 9.5 Hz, 1H), 7.50 (d, J = 8.4 Hz, 1H), 7.13 (d, J = 2.2 Hz, 1H), 7.07 (dd, J = 8.4, 2.2 Hz, 1H), 6.41 (d, J = 9.5 Hz, 1H), 2.35 (s, 3H).

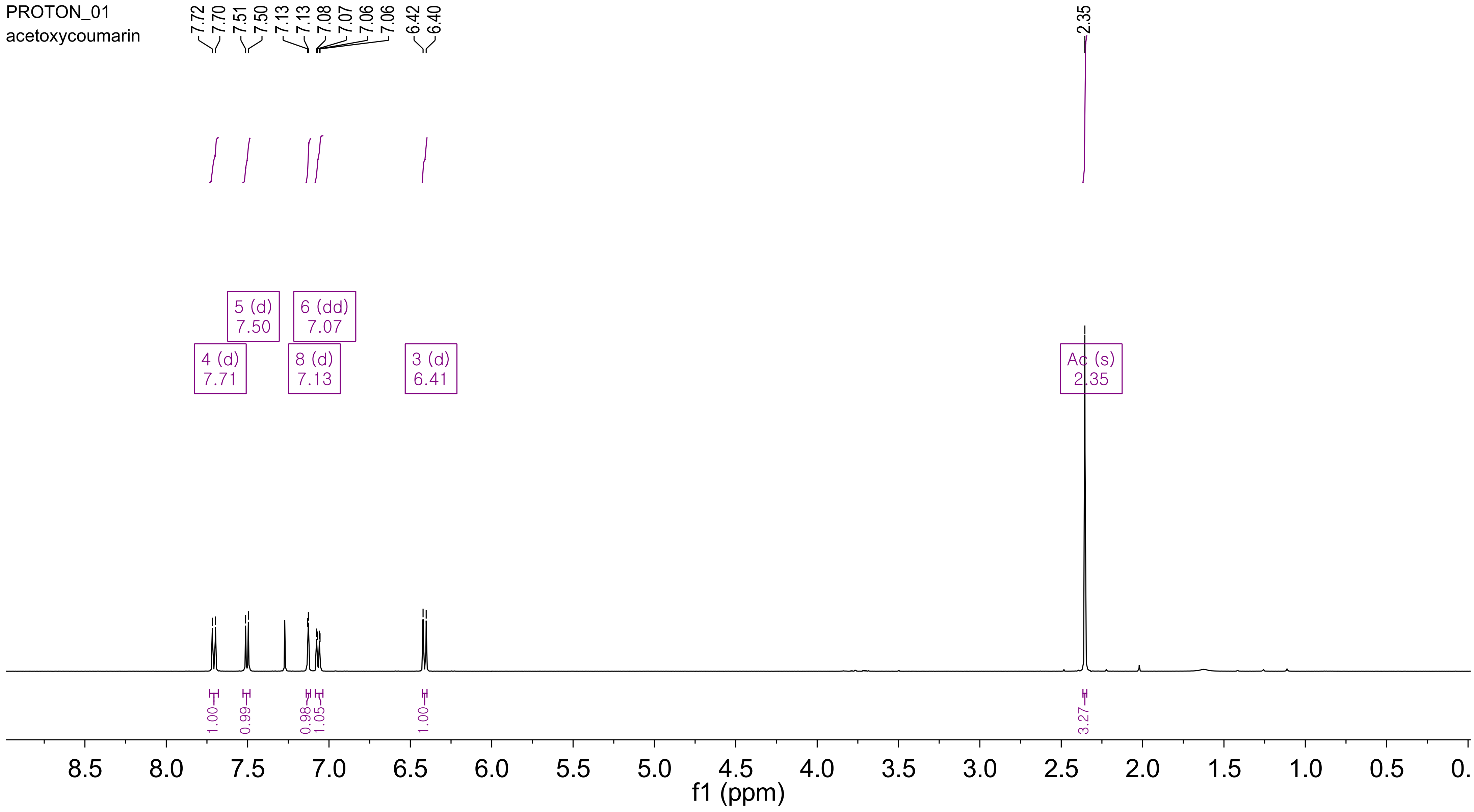

Supplement: Supplementary file 1 [file molecules-25-03124-s001.pdf]
